# Identification of a Biosynthetic Gene Cluster Responsible for the Production of a New Pyrrolopyrimidine Natural Product—Huimycin

**DOI:** 10.3390/biom10071074

**Published:** 2020-07-18

**Authors:** Hui Shuai, Maksym Myronovskyi, Suvd Nadmid, Andriy Luzhetskyy

**Affiliations:** 1 Pharmazeutische Biotechnologie, Universität des Saarlandes, 66123 Saarbrücken, Germany; hui.shuai@uni-saarland.de (H.S.); maksym.myronovskyi@uni-saarland.de (M.M.); suvdn@yahoo.com (S.N.); 2 School of Pharmacy, Mongolian National University of Medical Sciences, S. Zorig Street, 14210 Ulaanbaatar, Mongolia; 3 Helmholtz-Institut für Pharmazeutische Forschung Saarland, 66123 Saarbrücken, Germany

**Keywords:** kutzneria, nucleoside, pyrrolopyrimidines, secondary metabolites, heterologous expression

## Abstract

Pyrrolopyrimidines are an important class of natural products with a broad spectrum of biological activities, including antibacterial, antifungal, antiviral, anticancer or anti-inflammatory. Here, we present the identification of a biosynthetic gene cluster from the rare actinomycete strain *Kutzneria albida* DSM 43870, which leads to the production of huimycin, a new member of the pyrrolopyrimidine family of compounds. The huimycin gene cluster was successfully expressed in the heterologous host strain *Streptomyces albus* Del14. The compound was purified, and its structure was elucidated by means of nuclear magnetic resonance spectroscopy. The minimal huimycin gene cluster was identified through sequence analysis and a series of gene deletion experiments. A model for huimycin biosynthesis is also proposed in this paper.

## 1. Introduction

Pyrrolopyrimidines are a class of natural products characterized by the presence of a 7-deazapurine moiety in their structures [1]. This class of compounds is widely spread among three domains of life: archaea, bacteria, and eukarya [1]. The study of pyrrolopyrimidines began in 1956 with the discovery of an anti-Candida compound, toyocamycin, in the culture broth of *Streptomyces toyocaensis* [2]. Soon after the discovery of toyocamycin, other secreted 7-deazapurine compounds, tubercidin and sangivamycin, were isolated from the culture filtrates of *Streptomyces tubercidicus* and *Streptomyces rimosus* [3,4]. To date, more than 30 different 7-deazapurines have been isolated from various biological sources, including bacteria, cyanobacteria, red algae, marine sponges, and tunicates [1]. In addition to secreted pyrrolopyrimidines, which act as natural products, pyrrolopyrimidines can also be found as modified bases in tRNA. Queuosine is one of the most studied modified nucleosides that contains a 7-deazapurine moiety [5]. It is present in the wobble position of aspartyl, asparaginyl, histidyl and tyrosyl tRNA, where it likely modifies base pairing characteristics of tRNA and enhances translational efficiency [6,7,8]. Distributed ubiquitously among prokaryotes and eukaryotes, queuosine cannot be found in archaebacteria. In archaeal species, archeosine, a noncanonical 7-deazapurine nucleoside, is incorporated into tRNA [9].

Secreted pyrrolopyrimidines often possess antibacterial, antifungal, anticancer, antiviral or anti-inflammatory activities [1,10,11,12]. Their structural similarity to purine bases allows 7-deazapurines to interfere with various cellular processes involving nucleotides and nucleosides [1]. For instance, toyocamycin and tubercidin have been shown to be substrates of mammalian adenosine kinase [13]. In the phosphorylated form, toyocamycin is recognized by mammalian DNA and RNA polymerases and can become incorporated into DNA and RNA [14]. Although pyrrolopyrimidines are not substrates for amino acid aminoacylation, toyocamycin and sangivamycin inhibit this process [15]. When incorporated into the acceptor stem of tRNA, pyrrolopyrimidine inhibits tRNA aminoacylation [15]. Sangivamycin has recently been demonstrated to inhibit protein kinases [16,17]. A broad range of biological activities characteristic of pyrrolopyrimidines makes them promising potential drug leads for the pharmaceutical industry. Recent U.S. food and drug administration (FDA) approval of ribociclib and baricitinib, which both contain a 7-deazapurine moiety, for the treatment of breast cancer and rheumatoid arthritis further emphasizes the pharmaceutical importance of the pyrrolopyrimidine class of compounds [18,19].

In this study, we report the identification and heterologous expression of a biosynthetic gene cluster of *Kutzneria albida* DSM 43870 that encodes a new member of the pyrrolopyrimidine family of natural products, huimycin. The compound was isolated, and its structure was elucidated using extensive 2D nuclear magnetic resonance spectroscopy (NMR). Through a series of DNA deletion experiments, the minimal cluster required for huimycin biosynthesis was determined. Based on the cluster analysis, we also propose the biosynthetic route that produces huimycin.

## 2. Materials and Methods

### 2.1. Strains and BACs

All of the strains used in this study are listed in Table S1. The BACs are listed in Table S2. *Escherichia coli* strains were cultured in LB medium [20]. For sporulation and conjugation, *Streptomyces* strains were cultivated on soya flour mannitol agar (MS agar) [21] and in liquid tryptic soy broth (TSB; Sigma-Aldrich, St. Louis, MO, USA). DNPM medium was used for secondary metabolite production liquid [22]. The antibiotics ampicillin, kanamycin, apramycin, hygromycin and nalidixic acid were supplemented when required.

### 2.2. DNA Manipulation

Isolation of DNA and all subsequent manipulations were performed according to standard protocols [20]. BAC extraction from the genomic library of *K. albida* was performed using the BACMAX™ DNA purification kit (Lucigen, Middleton, WI, USA). Restriction endonucleases were used according to manufacturer’s recommendations (New England Biolabs, Ipswich, MA, USA). All of the primers used in this study are listed in Table S3.

To determine the minimal huimycin biosynthetic cluster a series of BACs with the deletions of single and multiple genes was constructed. In the BACs 2I16_LS and 2I16_RS large DNA regions upstream and downstream of the putative huimycin cluster were deleted. In the BACs 2I16_4069, 2I16_4074, 2I16_4075, and 2I16_4076, the genes KALB_4069, KALB_4074, KALB_4075, and KALB_4076 were inactivated. In the BAC 2I16_LS 20 kb DNA fragment upstream of the *huiA* gene was deleted. For this purpose, the hygromycin gene was amplified from the pACS-hyg plasmid with the LS-F/LS-R pair of primers. The obtained PCR fragment was used for the Red-ET modification of the BAC 2I16 [23]. The constructed BAC 2I16 was checked by restriction mapping (Figure S1) and PCR with the LS_chF/LS_chR pair of primers with subsequent sequencing of the obtained PCR product.

The BACs 2I16_RS, 2I16_4069, 2I16_4074, 2I16_4075 and 2I16_4076 were constructed in a similar manner. Here, ampicillin resistance marker was used for recombineering purposes instead of hygromycin marker. The marker was amplified from the pUC19 plasmid with the pairs of primers RS_F/RS_R, 4069_F/4069_R, 4074_F/4074_R, 4075_F/4075_R, and 4076_F/4076_R. The obtained PCR products were utilized for the construction of the abovementioned BACs using Red-ET modification. The construction of the BACs was confirmed using restriction mapping (Figure S1), PCR with the pairs of primers RS_chF/RS_chR, 4069_chF/4069_chR, 4074_chF/4074_chR, 4075_chF/4075_chR, 4076_chF/4076_chR, and sequencing.

### 2.3. Metabolite Extraction and Analysis

*Streptomyces albus* strains containing 2I16 BAC and its derivatives were grown in 15 mL of TSB medium for 24 h. One mL of the seed culture was used for inoculation of 100 mL of DNPM medium. The cultures were cultivated for seven days at 28 °C. Huimycin was extracted with the equal amount of butanol from the culture supernatant, evaporated, and dissolved in methanol.

For mass determination a Bruker Amazon Speed and a Thermo LTQ Orbitrap XL mass spectrometer were used. Both machines were coupled to UPLC Thermo Dionex Ultimate3000 RS. Analytes were separated on a Waters ACQUITY BEH C18 Column (1.7 μm, 2.1 mm × 100 mm) with water +0.1% formic acid and acetonitrile +0.1% formic acid as the mobile phase.

### 2.4. Huimycin Isolation and NMR Data Acquisition

*Streptomyces albus* 2I16 was cultivated into 20 L of DNPM medium at 28 °C for seven days. The mycelial part was separated by centrifugation. The supernatant was extracted once with the equal amount of butanol. The solvent was evaporated under vacuum in a rotary evaporator. The huimycin was purified using size-exclusion and reverse phase chromatography. Size-exclusion chromatography was performed using Sephadex LH-20 (GE Healthcare, USA) and methanol as a solvent. The HPLC separation was performed on semipreparative HPLC (Dionex UltiMate 3000, Thermo Fisher Scientific, USA) equipped with a C18 column (Synergi 10 μm, 250 × 10 mm; Phenomenex, Aschaffenburg, Germany). Water +0.1% formic acid and acetonitrile +0.1% formic acid were used as the mobile phase.

NMR spectra were recorded in meod4 at 500 MHz on a Bruker Avance 500 spectrometer (Bruker, BioSpin GmbH, Rheinstetten, Germany) equipped with a 5 mm TXI cryoprobe. HSQC, HMBC, ^1^H-^1^H COSY, and 2D TOCSY experiments were acquired using standard pulse program. CNST13 of HMBC were set as 2,3JC-H = 2,8 and 10 Hz.

### 2.5. Genome Mining and Bioinformatics Analysis

The *K. albida* genome was screened for secondary metabolite biosynthetic gene clusters using the antiSMASH online tool [24]. Geneious R9 (Biomatters Ltd.) software package was used for DNA sequence analysis.

## 3. Results and Discussion

### 3.1. Identification of the Huimycin Gene Cluster Through Its Heterologous Expression in Streptomyces albus Del14

**Figure 1:**
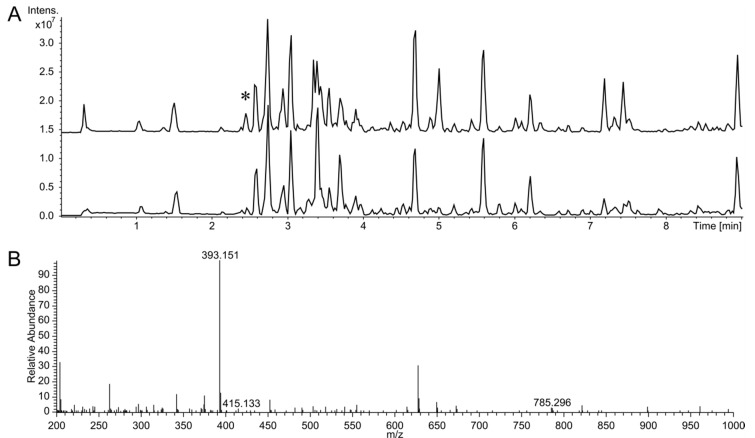
Liquid chromatography–mass spectrometry detection of huimycin. (**A**) Base peak chromatograms of crude extracts of *S. albus* 2I16 harboring the huimycin gene cluster (upper chromatogram) and of the control strain *S. albus* Del14 (lower chromatogramm). The new peak found in the *S. albus* 2I16 extract is indicated with an asterix. (**B**) High resolution mass spectrum of the new peak corresponding to huimycin. The molecular ion peaks at m/z 393.151, 415.133 and 785.296 correspond to huimycin [M + H]+, its sodium adduct [M + Na]+ and its dimer [2M + H]^+^.

Recently, we reported the complete genome sequence of *Kutzneria albida* DSM 43870 (GenBank accession number NZ_CP007155), which is a representative of a rarely observed genus in the *Pseudonocardiaceae* family [25]. Forty-six putative clusters encoding secondary metabolites were identified in the genome of this strain [25]. To enable analysis of these metabolites, a genomic library of *K. albida* was constructed using an integrative BAC vector. In the course of systematic activation of cryptic secondary metabolite clusters from *K. albida*, a cluster annotated by the antiSMASH genome mining software as “nucleoside biosynthetic cluster” was expressed in the heterologous host strain *S. albus* Del14. For this purpose, a BAC 2I16 vector containing the cluster was isolated from the constructed genomic library and transferred into the chassis strain *S. albus* Del14 by conjugation. The obtained exconjugant strain *S. albus* 2I16 and the corresponding control strain without the BAC *S. albus* Del14 were fermented in the production medium DNPM. The culture filtrate of the strains was extracted with butanol, and the extracts were analyzed by liquid chromatography–mass spectrometry (LC-MS). This analysis revealed a new peak in the extract of *S. albus* 2I16 as a result of the cluster expression (Figure 1). Subsequent analysis of the extract using high resolution LC-MS revealed that the identified peak corresponded to a compound with an [M + H]+ of 393.15 m/z (Figure 1). The extract of the control strain *S. albus* Del14 did not contain the identified ion. A search in a natural product database for the identified high-resolution mass did not generate any matches, implying that the identified compound might be new.

### 3.2. Isolation and Structure Elucidation of the Huimycin

To obtain structural information about the potentially new compound obtained from the heterologous expression of the nucleoside gene cluster, we set out to purify it. For this purpose, the *S. albus* 2I16 strain with the expressed nucleoside gene cluster was inoculated into 20 L of DNPM medium, and the culture broth was extracted with butanol. The compound was separated from contaminants in the extract using size-exclusion and reverse-phase chromatography. A total of 8.2 mg of the compound was isolated during the purification process and was used for subsequent structure elucidation purposes (Figures S2–S9).

The combination of the ^1^H NMR spectrum with the HSQC data indicated an isolated aromatic signal, one anomeric methine, one methoxy signal together with three oxygenated methines and one methylene signal as well as a methyl group. The TOCSY and COSY spectra suggested the presence of a sugar moiety, which was determined to be 1-amino-2-deoxy-glucose by means of analyzing HMBC cross peaks. HMBC correlations from H-2′ and H-9′ to C-8′ further revealed the substitution of an acetate on the amino group of the sugar (Table 1). The large coupling constant observed for the anomeric proton of the glucose moiety at 5.28 (d, J = 9.6 Hz) suggested its β-orientation. Further ROESY correlations were observed between H1′/H3′/H5′ as well as H2′/H4′ of the glucose moiety.

The constitution of the aglycone core was determined to be a purine bearing substitutions at C-2, C-6 and C-7. Several HMBC experiments with different long-range coupling constants (CNST13) were used to link the substitutions to the correct positions. The HMBC spectrum acquired with CNST13 = 2 Hz showed a more intense cross peak from the H-8 to a carbon at δ 160.3 than what was observed in the HMBC with 10 Hz data, suggesting that the carbon at δ 160.3 (C-2) is the furthest carbon from H-8 and shows a five bond correlation. Furthermore, according to HMBC, the alpha proton of the sugar moiety showed correlation with C-2, indicating its linkage. The methoxy group was found to be linked on C-6 because of the HMBC correlation (in CNST13 = 2 Hz data) of methyl protons to C-5 (at δ 98.7 ppm), showing a four-bond heteronuclear correlation. Due to the biosynthetic similarity of this molecule to toyocamycin and their NMR data similarity, the cyano-group was deduced to be attached to C-7. This was supported by the HMBC correlation from H-8 to carbon at δ 116.3 (C-10) and the chemical shift of C-7 at δ 84.5 ppm.

**Figure 2:**
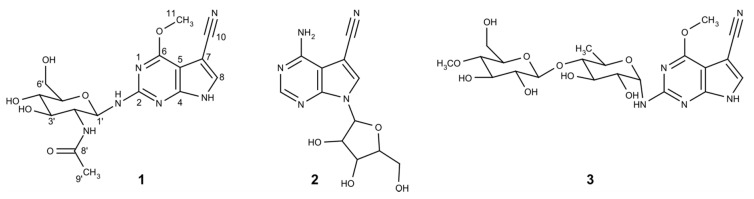
The structures of (**1**) huimycin, (**2**) toyocamycin, and (**3**) dapiramicin A.

Finally, the planar structure was deduced, as shown in Figure 2, which is in concordance with the HR-ESI-MS data (measured 393.1509, calculated 393.1517, Δppm 2.0). The isolated compound was named huimycin and was identified as a novel natural compound. Structurally, huimycin is closely related to dapiramicins A and B, differing from the latter only in the sugar moiety attached to the aglycone (Figure 2) [26,27].

### 3.3. Determination of the Minimal Huimycin Gene Cluster

The presence of a 7-deazapurine moiety, typical of a pyrrolopyrimidine class of nucleoside antibiotics [1], in the structure of the isolated huimycin indicates the participation of the expressed nucleoside cluster in its production. The huimycin gene cluster was expressed in the heterologous host strain as a part of the large 95-kb chromosomal fragment in BAC 2I16. To determine the minimal set of genes required for huimycin biosynthesis, a sequence analysis and a series of gene deletion experiments were performed.

**Figure 3:**
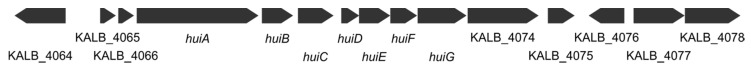
The chromosomal fragment of *K. albida* containing the huimycin biosynthetic gene cluster.

The sequence analysis of the DNA fragment cloned in BAC 2I16 leading to nucleoside production revealed the presence of seven open reading frames, *huiA – huiG* (locus tags KALB_4067 – KALB_4073), which are highly likely to be involved in huimycin biosynthesis (Figure 3, Table 2). Five of these genes shared homology at the protein level with the genes within the biosynthetic cluster of toyocamycin—the parent compound of the pyrrolopyrimidine class of antibiotics (Figure 2, Table 2) [28]. The *huiA* gene, encoding a putative transcriptional regulator, shares homology with the regulatory gene *toyA* [28]. The genes *huiB*, *huiD*, *huiE* and *huiF* encode putative 7-cyano-7-deazaguanine synthase, 6-carboxytetrahydropterin synthase, 7-carboxy-7-deazaguanine synthase and GTP cyclohydrolase I, and they share homology with the toyocamycin biosynthetic genes *toyM*, *toyB*, *toyC* and *toyD*, respectively (Table 2) [28]. The structural similarities between huimycin and toyocamycin also imply similar biosynthetic routes leading to the production of antibiotics. The genes *huiC* and *huiG* do not have counterparts in the toyocamycin gene cluster; they encode putative SAM-dependent methyltransferase and glycosyltransferase, respectively (Table 2). The genes *huiC* and *huiG* are likely responsible for the structural differences between huimycin and toyocamycin.

The gene *huiA* encodes a transcriptional regulator and was assumed to constitute the 5′ outer border of the huimycin cluster (Figure 3). The counterpart of the gene *huiA* within the toyocamycin biosynthetic pathway, *toyA*, also constitutes the first gene of the cluster [28]. The deletion of the *toyA* gene completely abolishes toyocamycin production [29]. The first three genes in the region upstream of the *huiA* gene, KALB_4064, KALB_4065, and KALB_4066 (Figure 3), encode an enhanced intracellular survival protein and two hypothetical proteins, respectively. No function in nucleoside biosynthesis could be assigned to these three genes. This further strengthens the assumption that the *huiA* gene constitutes the 5′ boundary of the cluster. To corroborate this assumption empirically, the 20 kb DNA fragment upstream of the *huiA* gene was deleted within the 2I16 BAC through RedET recombineering. The obtained construct, 2I16_LS, resulted in huimycin production when it was transferred into an *S. albus* strain. This clearly indicated that the genes in the upstream region of the *huiA* are not required for huimycin biosynthesis. Thus, it is likely that the *huiA* gene is at the 5′ end of the huimycin gene cluster.

Prediction of the 3′ boundary of the huimycin cluster was not obvious from the gene annotation. The last gene showing homology to toyocamycin biosynthetic genes is *huiF* (Table 2). This gene is followed by the gene *huiG*, which encodes a glycosyltransferase that might participate in nucleoside biosynthesis. The genes KALB_4074, KALB_4075, KALB_4076, KALB_4077, and KALB_4078 in the downstream region of the *huiG* encode a putative carbamoyltransferase, pyridoxamine 5′-phosphate oxidase family protein, SAM-dependent methyltransferase, and two glycosyltransferases, respectively. As is evident in the huimycin structure (Figure 2), glycosyltransferase and methyltransferase activities are required to produce huimycin. The required methyltransferase may be encoded either by *huiC* or by KALB_4076. It is also not obvious which of the three glycosyltransferase genes, *huiG*, KALB_4077 or KALB_4078, participates in huimycin biosynthesis. To clarify this, the 36 kb DNA fragment downstream of the KALB_4076 gene was deleted in the 2I16 BAC. The obtained BAC 2I16_RS led to huimycin production when introduced into *S. albus*. This clearly indicates that the genes in the downstream region of KALB_4076, including the two glycosyltransferase genes KALB_4077 and KALB_4078, do not participate in huimycin biosynthesis. The possible participation of the genes KALB_4074, KALB_4075, and KALB_4076 in huimycin biosynthesis was assessed by their inactivation in the 2I16 BAC. The obtained BACs (2I16_4074, 2I16_4075 and 2I16_4076) contained the deletions of the genes KALB_4074, KALB_4075 and KALB_4076, respectively, and they resulted in huimycin production when introduced in *S. albus*. This shows that none of the inactivated genes is essential for huimycin production. Thus, it is likely that the *huiG* gene is at the 3′ end of the huimycin gene cluster.

Deletion of the genes located outside the *huiA – huiG* fragment have been shown not to affect the huimycin production in the heterologous strain *S. albus*. These genes are considered not to participate in huimycin biosynthesis, however a possibility exists that some of the deleted genes are crosscomplemented by one or several host genes. To demonstrate the importance of the *huiA–huiG* fragment for nucleoside production, the *huiC* gene, encoding the methyltransferase, was deleted from the 2I16 BAC. The deletion of the *huiC* gene completely abolished the production of the huimycin, showing its involvement in the production of this nucleoside.

### 3.4. Biosynthesis of Huimycin

Seven genes, *huiA–huiG*, have been shown to be sufficient for huimycin production. The *huiA* gene encodes a putative transcriptional regulator and is likely to be involved in the regulation of expression of the huimycin biosynthetic gene cluster. The homolog of *huiA*, the *toyA* gene, encodes a pathway-specific regulator of the toyocamycin gene cluster [28]. The six genes, *huiB–huiG*, encode structural enzymes that catalyze huimycin biosynthetic steps. The products of the genes *huiB*, *huiD*, *huiE*, and *huiF* display significant sequence similarity with the products of the toyocamycin biosynthetic genes *toyM*, *toyB*, *toyC*, and *toyD*, respectively. The genes *huiC* and *huiG* have no homologs in the toyocamycin cluster and encode a putative methyltransferase and glycosyltransferase, respectively. The considerable sequence similarity between huimycin and toyocamycin gene clusters implies the similarities in the biosynthetic routes leading to the production of the compounds.

**Figure 4:**
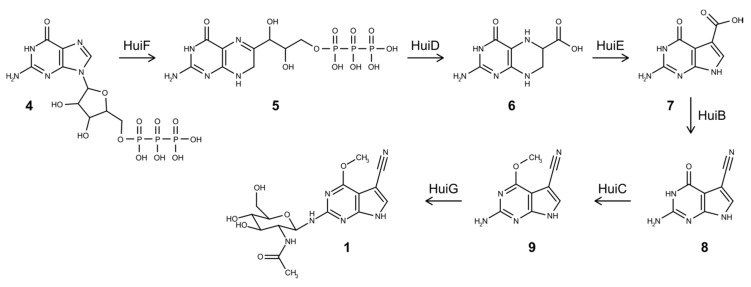
Proposed biosynthetic pathway of huimycin. (**4**) GTP, (**5**) 7,8-dihydroneopterin triphosphate, (**6**) 6-carboxy-5,6,7,8-tetrahydropterin, (**7**) 7-carboxy-7-deazaguanine, (**8**) 7-cyano-7-deazaguanine, (**9**) 2-amino-6-methoxy-7-cyano-7-deazapurine, and (**1**) huimycin.

Similar to toyocamycin, GTP is regarded as a main precursor for huimycin production [1,28]. The first reaction in huimycin biosynthesis is the conversion of GTP into 7,8-dihydroneopterin triphosphate (H2NTP) (Figure 4). This reaction is catalyzed by the product of the gene *huiF*, which encodes a putative GTP cyclohydrolase I. The product of the *huiF* gene shares 66% identity with the product of the *toyD* gene, which also catalyzes the first step of the toyocamycin biosynthesis [1,28]. The second step in the pathway is catalyzed by 6-carboxytetrahydropterin synthase encoded by *huiD*, which converts H2NTP into 6-carboxy-5,6,7,8-tetrahydropterin (CPH4) (Figure 4). The product of the *huiD* gene shares 65% identity with the product of *toyB*, which is responsible for the similar reaction in the toyocamycin biosynthesis [1,28]. The third biosynthetic step is catalyzed by the product of the *toyC* homolog, *huiE*, which encodes 7-carboxy-7-deazaguanine synthase. This enzyme converts CPH4 into 7-carboxy-7-deazaguanine (CDG) [1,28]. The products of *toyC* and *huiE* share 50% identity. The last step common for both huimycin and toyocamycin biosynthesis is the conversion of the CDG into 7-cyano-7-deazaguanine (PreQ0) through the action of 7-cyano-7-deazaguanine synthase (Figure 4). This enzyme is encoded by the *toyM* homolog, *huiB*. The protein products of these genes share 72% identity. We propose that the last two enzymatic steps required for the conversion of PreQ0 into huimycin are the methylation of 7-cyano-7-deazaguanine and the attachment of the N-acetylglucosamine moiety (Figure 4). The methylation reaction is likely to be catalyzed by the product of the gene *huiC*, which encodes a SAM-dependent methyltransferase. The attachment of the N-acetylglucosamine to the 2-amino-6-methoxy-7-cyano-7-deazapurine is catalyzed by the glycosyltransferase encoded by the last gene in the huimycin gene cluster – *huiG*. The order in which the last two reactions take place in the huimycin biosynthesis is not known. The isolation of dapiramicin B aglycone (2-amino-4-methoxy-5-cyanopyrrolo[2,3-d]pyrimidine) from the culture of *Streptomyces* sp. MK63-43F2 implies that that methyltransfer reaction precedes glycosylation [30]. Huimycin is thus far the only known member of the pyrrolopyrimidine class of compounds with an N-acetylglucosamine moiety in its structure. Additionally, the position of the N-glycosidic bond distinguishes huimycin from most of the 7-deazapurine nucleosides. Only in the structures of dapiramicin A and B, sugar moieties are also attached to the amino group at the second position of the 7-deazapurine chromophore (Figure 2) [26,27]. Furthermore, dapiramicin A and B share the same chromophore as huimycin. Since the dapiramicin biosynthetic gene cluster has not yet been discovered, it is not possible to study whether the structural similarities between huimycin and the dapiramicins are also reflected on the DNA sequence level. The identified huimycin biosynthetic genes can be used to screen sequenced genome databases to identify gene clusters potentially encoding pyrrolopyrimidines distinct from toyocamycin.

## 4. Conclusions

In this paper, we reported the identification and successful heterologous expression of the gene cluster responsible for the production of huimycin, a new member of the pyrrolopyrimidine family of nucleoside natural products. Huimycin features that are unique for nucleosides include an N-acetylglucosamine moiety attached to the 2-amino-6-methoxy-7-cyano-7-deazapurine core. The minimal set of huimycin biosynthetic genes was identified through a series of gene deletion experiments. The expression of the huimycin gene cluster in *S. albus* reported here is the first example of successful heterologous expression of a secondary metabolite pathway from the rare actinomycetal genus *Kutzneria*.

## Figures and Tables

**Table 1:** Nuclear magnetic resonance spectroscopy data of huimycin.

Position	*δ*_C_^a^	δ_H_ mult. (J, Hz) ^b^	HMBC ^c^
2	160.3		
4	155.4		
5	98.7		
6	164.5		
7	84.5		
8	131.0	7.60, s	2 ^e^, 4, 5, 6 ^e^, 7, 10
10	116.3		
11	54.2	4.05, s	5 ^e^, 6
1′	83.6	5.28, d 9.6	2, 2′, 3′, 5′
2′	56.1	3.87 ^d^	1′, 3′, 8′
3′	76.3	3.55, m	2′, 4′
4′	71.9	3.38 ^d^	5′, 6′
5′	79.2	3.39 ^d^	
6′ a	62.6	3.69, m	4′
6′ b		3.85 ^d^	
8′	174.5		
9′	22.7	1.96, s	8′

^a^ Acquired at 125 MHz, referenced to solvent signal meod4 at δ 49.15 ppm. ^b^ Acquired at 500 MHz, referenced to solvent signal meod4 at δ 3.31 ppm. ^c^ Proton showing HMBC correlation to indicated carbons. ^d^ Overlapped signals. ^e^ Observed in HMBC with CNST13 = 2 Hz.

**Table 2:** Proposed functions of the genes in the DNA fragment containing huimycin gene.

Gene	Proposed Function	Homolog in Toy Pathway	Identities/Positives
KALB_4064	Enhanced intracellular survival protein		
KALB_4065	Hypothetical protein		
KALB_4066	Hypothetical protein		
*huiA*; KALB_4067	Pathway-specific regulator	*toyA*	33%/46%
*huiB*; KALB_4068	7-cyano-7-deazaguanine synthase	*toyM*	72%/82%
*huiC*; KALB_4069	SAM-dependent methyltransferase		
*huiD*; KALB_4070	6-carboxytetrahydropterin synthase	*toyB*	65%/76%
*huiE*; KALB_4071	7-carboxy-7-deazaguanine synthase	*toyC*	50%/55%
*huiF*; KALB_4072	GTP cyclohydrolase I	*toyD*	66%/75%
*huiG*; KALB_4073	Glycosyltransferase		
KALB_4074	Carbamoyltransferase		
KALB_4075	Pyridoxamine 5′-phosphate oxidase		
KALB_4076	SAM-dependent methyltransferase		
KALB_4077	Glycosyltransferases		
KALB_4078	Glycosyltransferases		

## Supplementary Materials

The following are available online at https://www.mdpi.com/2218-273X/10/7/1074/s1, Figure S1: Restriction mapping of the BAC 2I16 and its derivatives with gene deletions, Figure S2: ^1^H NMR (500 MHz, MeOD4) spectrum of huimycin 1, Figure S3: HSQC (500 MHz, MeOD4) spectrum of huimycin 1, Figure S4: HMBC (500 MHz, MeOD4) spectrum (CNST13 = 2 Hz) of huimycin 1, Figure S5: HMBC (500 MHz, MeOD4) spectrum (CNST13 = 6 Hz) of huimycin 1, Figure S6: HMBC (500 MHz, MeOD4) spectrum (CNST13 = 10 Hz) of huimycin 1, Figure S7: ^1^H-^1^H COSY (500 MHz, MeOD4) spectrum of huimycin 1, Figure S8: TOSCY (500 MHz, MeOD4) spectrum of huimycin 1, Figure S9: ROESY (500 MHz, MeOD4) spectrum of huimycin 1, Table S1: Bacterial strains used in this work, Table S2: Plasmids and BACs used in this work, Table S3: Primers used in this study.
