# Supplementary material for: Identification of a Biosynthetic Gene Cluster Responsible for the Production of a New Pyrrolopyrimidine Natural Product—Huimycin
Source: Biomolecules. 2020 Jul 18;10(7):1074. doi: 10.3390/biom10071074 (PMC7439116; doi:10.3390/biom10071074)
Supplement: Supplementary file 1 [file biomolecules-10-01074-s001.pdf]

biomolecules

Supplementary Information

Identification of a Biosynthetic Gene Cluster Responsible for the Production of a New Pyrrolopyrimidine Natural Product—Huimycin

Hui Shuai [1], Maksym Myronovskyi [1], Suvd Nadmid [1,2] and Andriy Luzhetskyy [1,3,*]

[1] Pharmazeutische Biotechnologie, Universität des Saarlandes, 66123 Saarbrücken, Germany; hui.shuai@uni-saarland.de (H.S.); maksym.myronovskyi@uni-saarland.de (M.M.)

[2] School of Pharmacy, Mongolian National University of Medical Sciences, S. Zorig street, 14210 Ulaanbaatar, Mongolia; suvdn@yahoo.com (S.N.)

[3] Helmholtz-Institut für Pharmazeutische Forschung Saarland, 66123 Saarbrücken, Germany

* Correspondence: a.luzhetskyy@mx.uni-saarland.de; Tel.: +49-0681-70223

Received: 21 June 2020; Accepted: 16 July 2020; Published: 12 August 2020

Figures

A

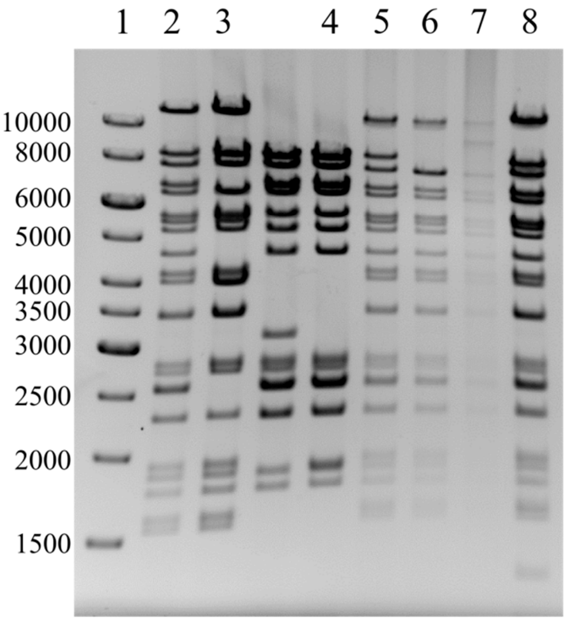

B

2I16	2I16_LS	2I16_RS	2I16_4069	2I16_4074	2I16_4075	2I16_4076
11195	11195	8213	11195	11195	11195	11195
8213	8213	7692	8433	7692	9609	8213
7692	7692	6794	7692	7638	7692	7692
6794	6505	6505	6794	6794	6794	6794
6505	5685	5685	6505	6505	6505	6505
5685	5481	5216	5685	5685	5685	5685
5481	5216	4617	5481	5481	5481	5481
5216	4181	2802	5216	5216	5216	5216
4617	4028	2700	4617	4617	4617	4617
4181	3979	2558	4181	4181	4181	4181
4028	3480	2556	4028	4028	4028	4028
3480	3429	2530	3480	3480	3480	3480
3429	2802	2291	3429	3429	3429	3429
2802	2700	2271	2802	2802	2802	2802
2700	2291	1882	2700	2700	2700	2700
2558	1920	1850	2558	2558	2558	2558
2556	1850	1748	2556	2556	2556	2556
2530	1748	1014	2530	2530	2530	2530
2291	1598	906	2291	2291	2291	2291
2271	1542	879	2271	2271	2271	2271
1920	1014	835	1920	1920	1920	1920
1850	906	811	1850	1850	1850	1850
1748	879	344	1748	1748	1748	1748
1598	344	22	1598	1598	1598	1598
1542	22		1542	1542	1542	1542
1014			1014	1014	1014	1249
906			906	906	906	1014
879			879	879	835	879
835			835	835	811	835
811			811	811	344	811
344			344	344	22	344
22			22	22		22

Figure S1. Restriction mapping of the BAC 2I16 and its derivatives with gene deletions. (**A**) Gel electrophoresis of KpnI digests of the following BACs: 2—2I16, 3—2I16_LS, 4—2I16_RS, 5—2I16_4069, 6—2I16_4074, 7—2I16_4075, 8—2I16_4076, 1—DNA ladder. Size of individual fragments in base pairs is shown. (**B**) The expected length of DNA fragments in base pairs obtained after KpnI digestion of the following BACs: 2I16, 2I16_LS, 2I16_RS, 2I16_4069, 2I16_4074, 2I16_4075, and 2I16_4076.

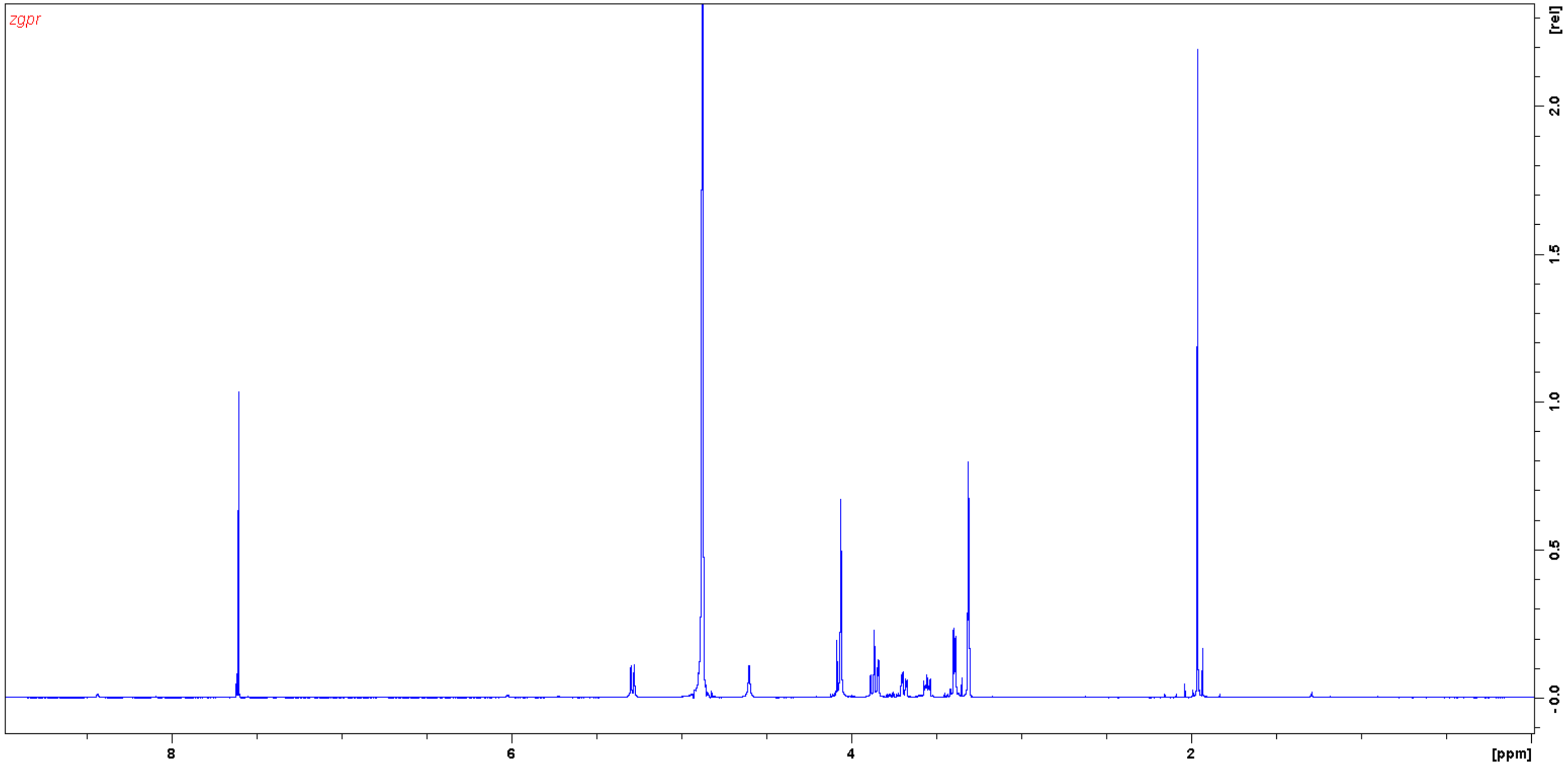

Figure S2. ^{1}H NMR (500 MHz, MeOD₄) spectrum of huimycin **1.**

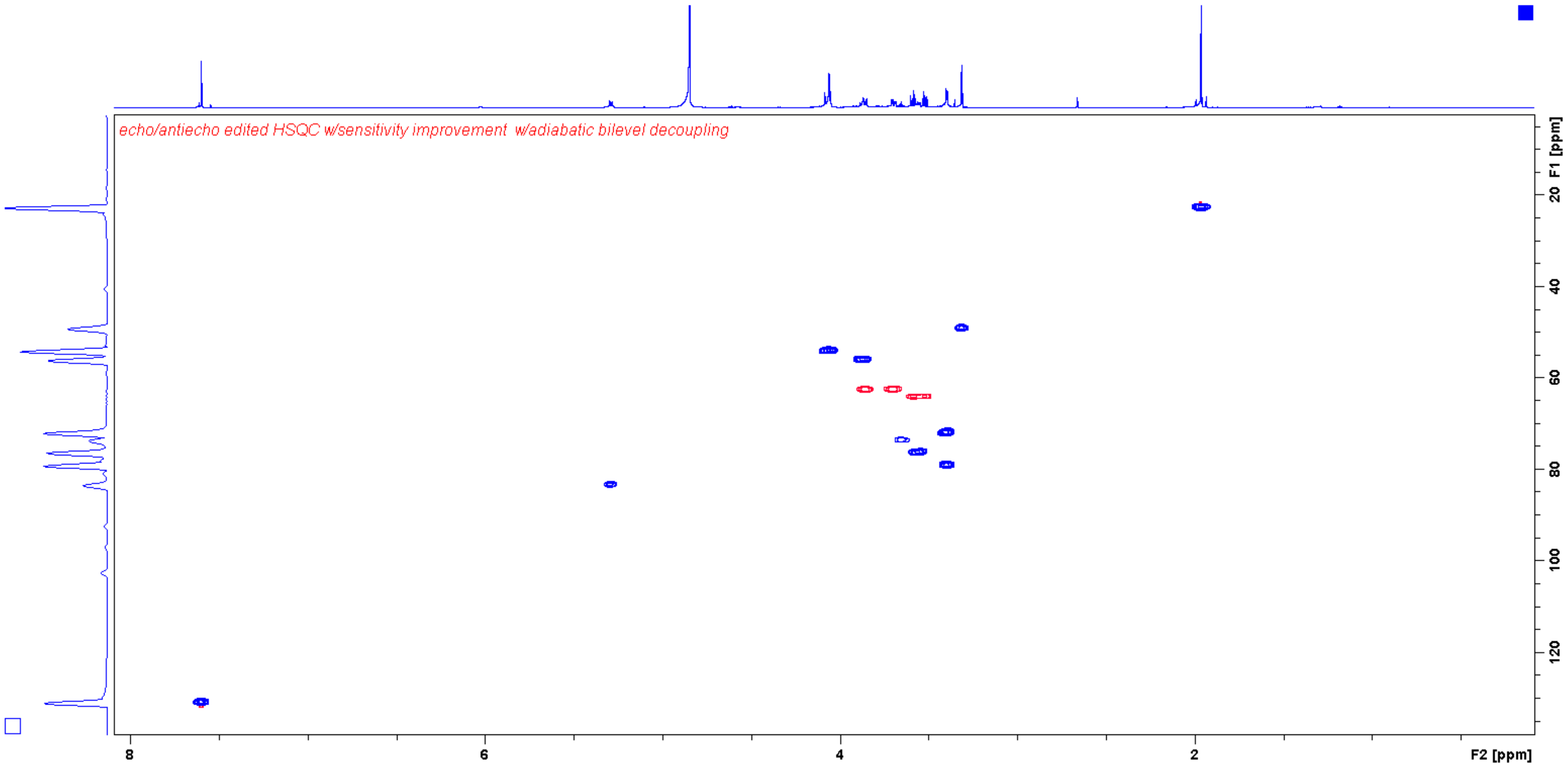

Figure S3. HSQC (500 MHz, MeOD4) spectrum of huimycin **1.**

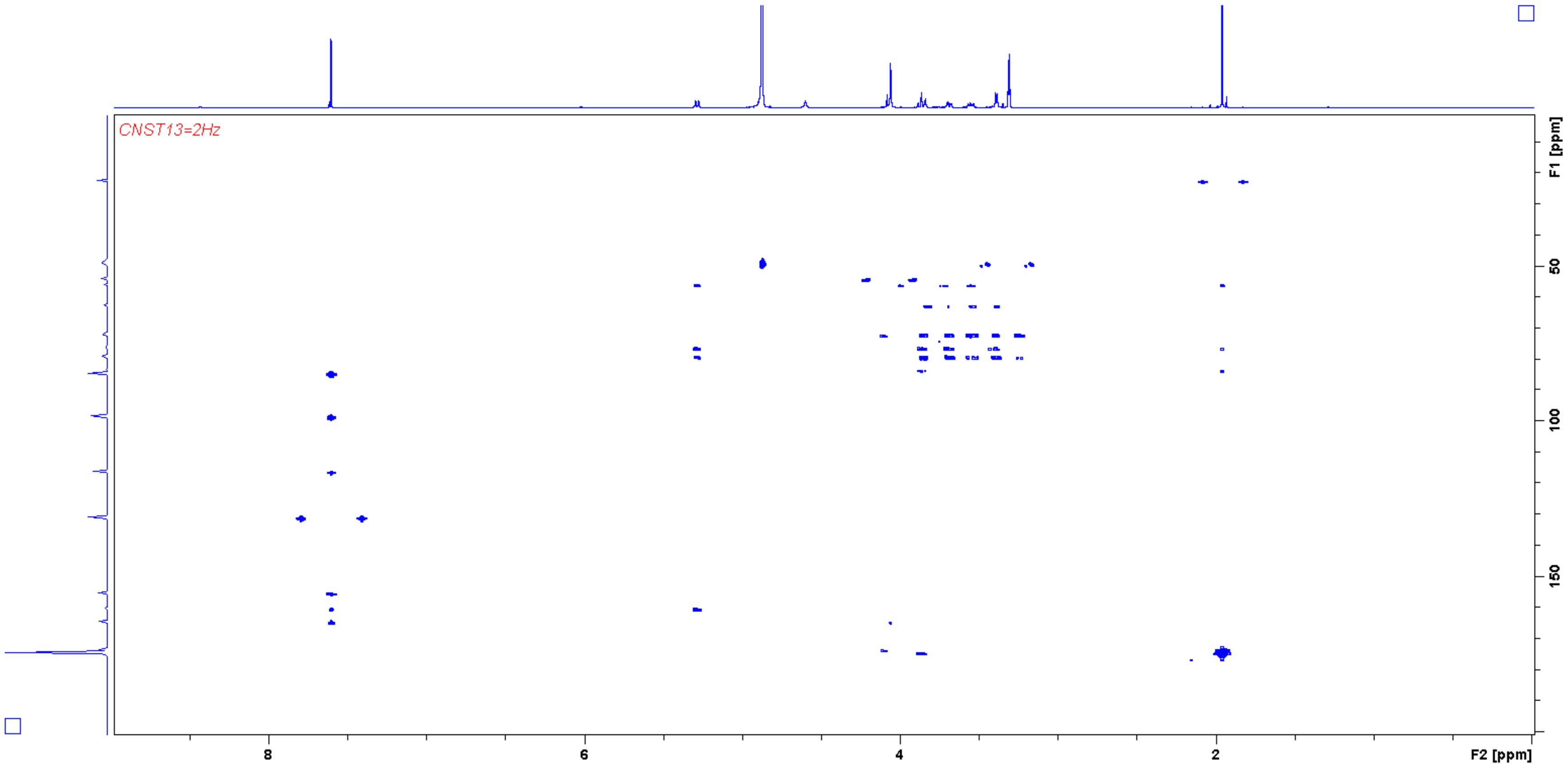

Figure S4. HMBC (500 MHz, $MeOD_4$) spectrum (CNST 13=2 Hz) of huimycin **1.**

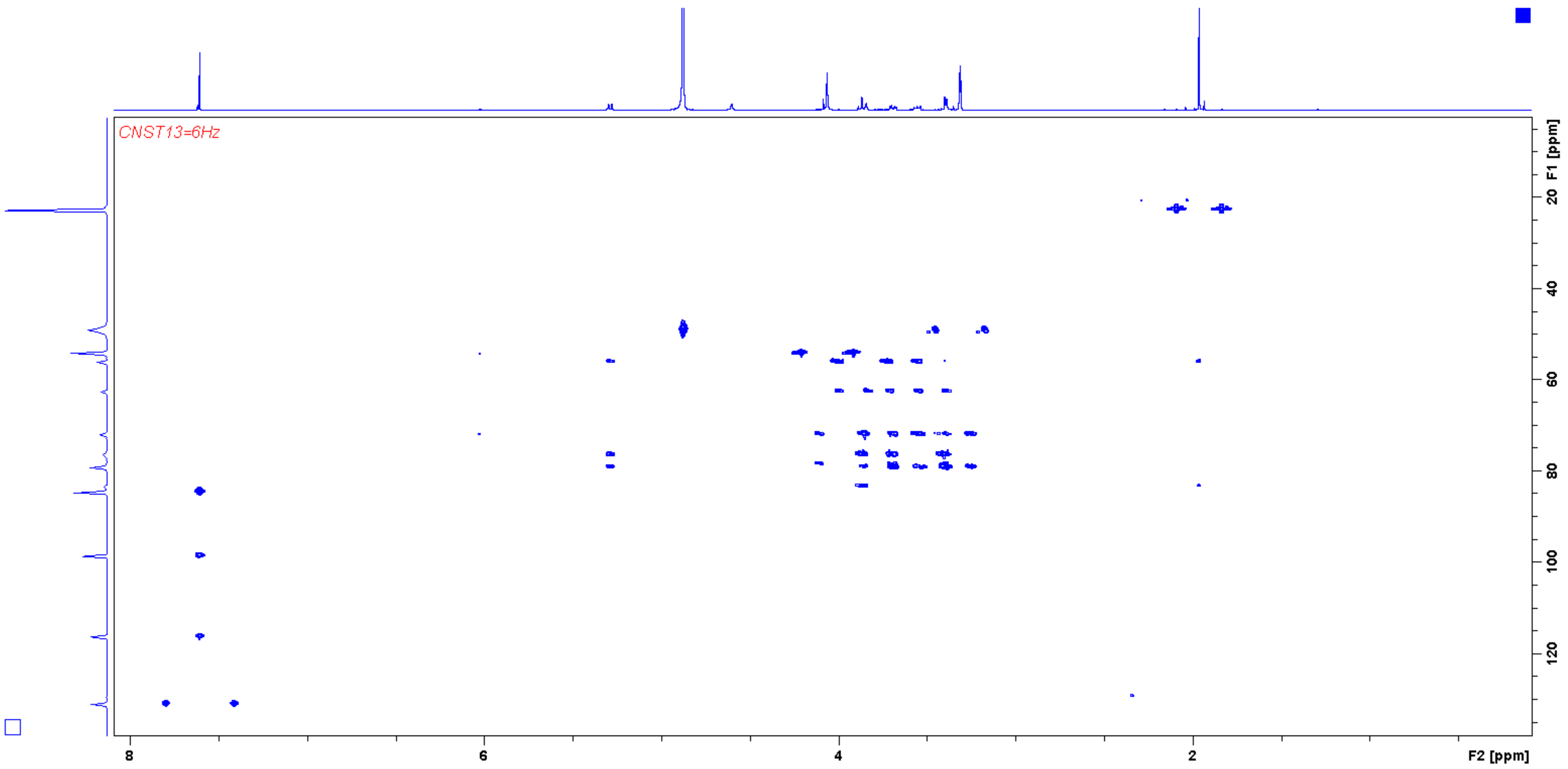

Figure S5. HMBC (500 MHz, $MeOD_4$) spectrum (CNST 13=6 Hz) of huimycin **1.**

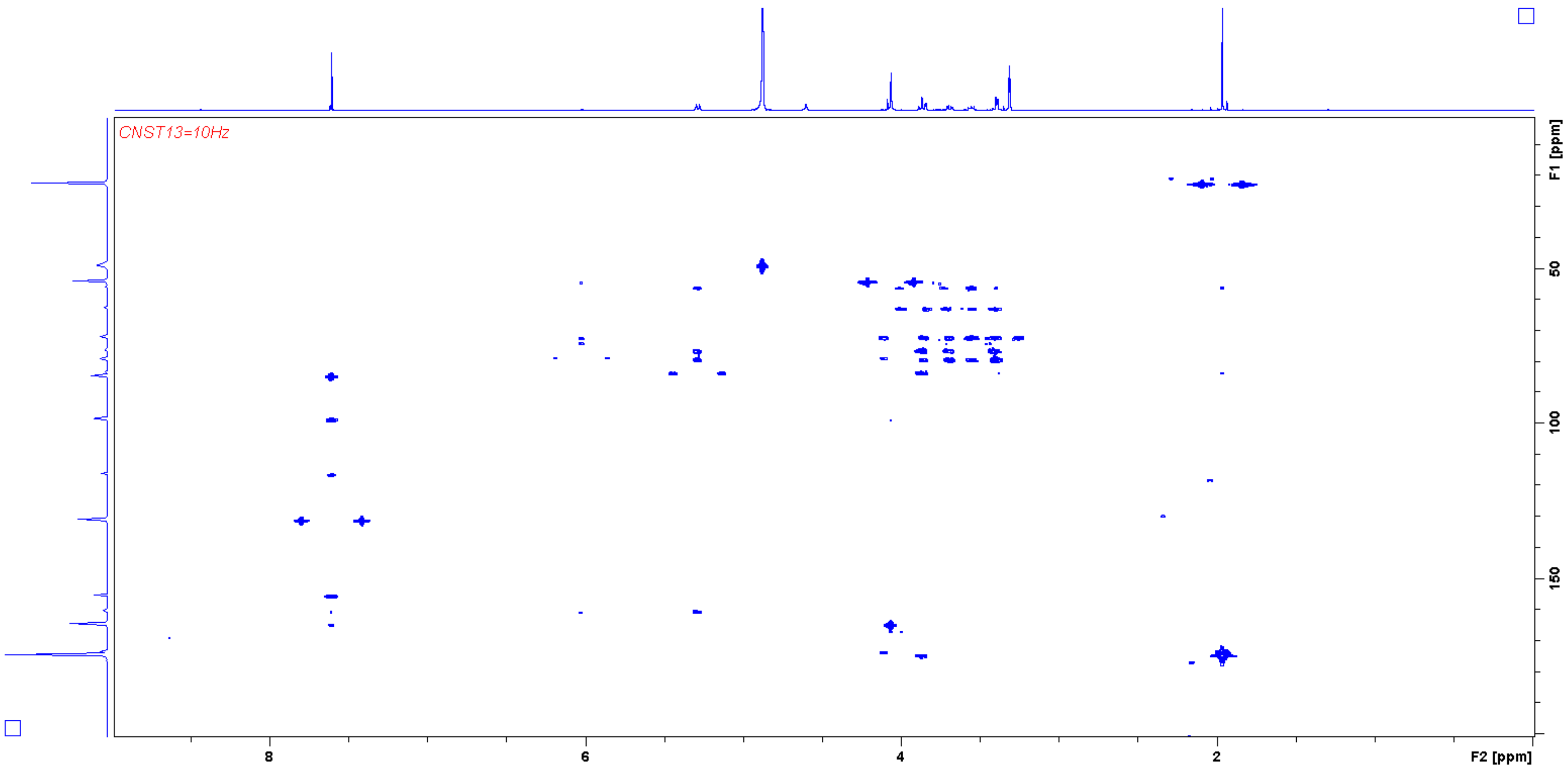

Figure S6. HMBC (500 MHz, MeOD4) spectrum (CNST 13=10 Hz) of huimycin **1.**

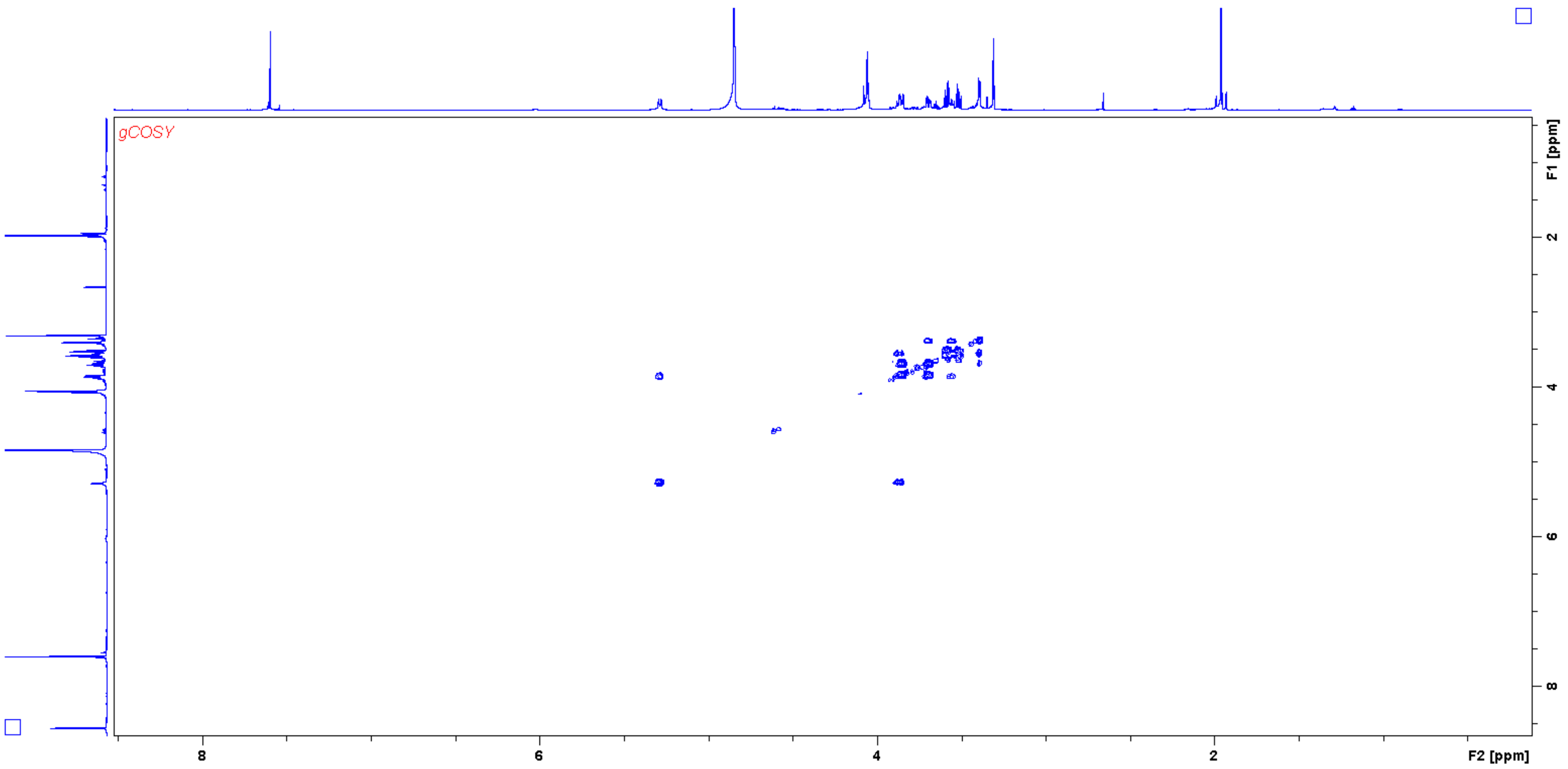

Figure S7. ^1H-^1H COSY (500 MHz, MeOD4) spectrum of huimycin **1.**

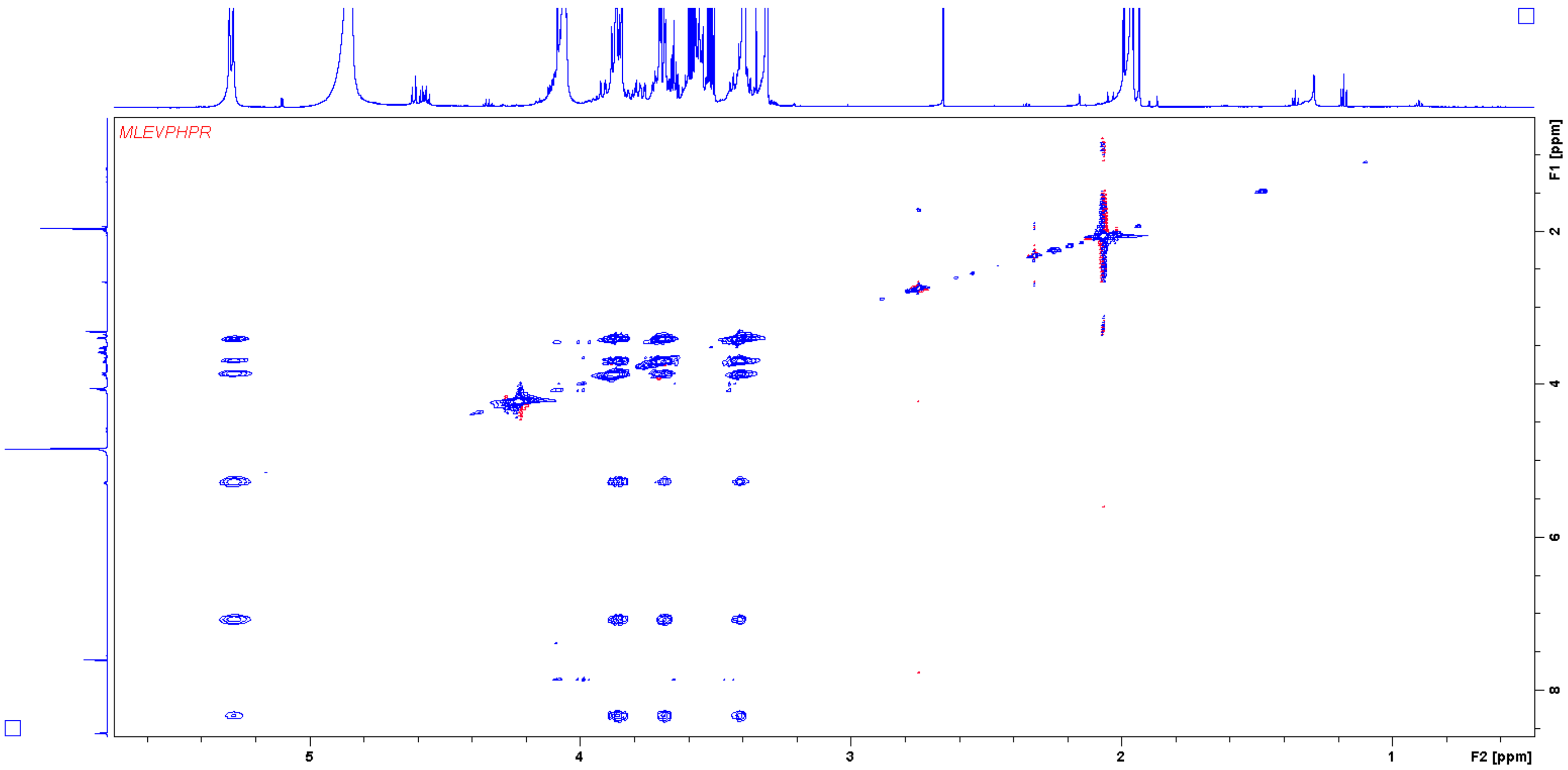

Figure S8. TOSCY (500 MHz, $MeOD_4$) spectrum of huimycin **1.**

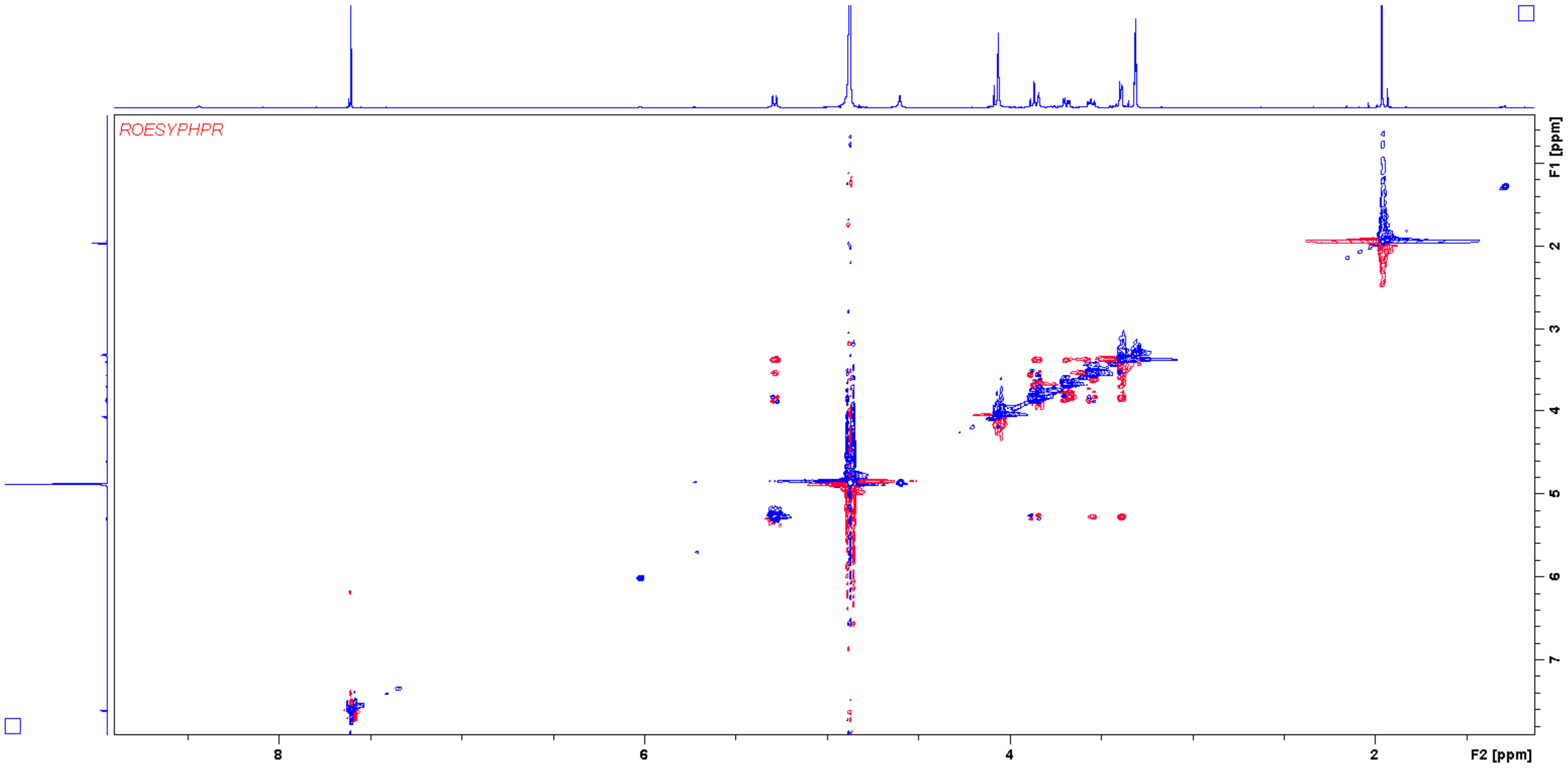

Figure S9. ROESY (500 MHz, MeOD4) spectrum of huimycin **1.**

Tables

Table S1. Bacterial strains used in this work.

Strain	Description	Reference or source
Kutzneria albida DSM 43870	The wild type strain; the source of the huimycin luster	[1]
Streptomyces albus Del14	The heterologous host strain; cluster-free derivative of the *S. albus* J1074	[2]
S. albus 2I16	Derivative of *S. albus* Del14 harboring the 2I16 BAC	This work
S. albus 2I16_LS	Derivative of *S. albus* Del14 harboring the 2I16_LS BAC	This work
S. albus 2I16_RS	Derivative of *S. albus* Del14 harboring the 2I16_RS BAC	This work
S. albus 2I16_4069	Derivative of *S. albus* Del14 harboring the 2I16_4069 BAC	This work
S. albus 2I16_4074	Derivative of *S. albus* Del14 harboring the 2I16_4074 BAC	This work
S. albus 2I16_4075	Derivative of *S. albus* Del14 harboring the 2I16_4075 BAC	This work
S. albus 2I16_4076	Derivative of *S. albus* Del14 harboring the 2I16_4076 BAC	This work
Escherichia coli ET12567 pUB307	Donor strain for intergeneric conjugation	[3]
Escherichia coli DH10β	General cloning strain	[4]

Table S2. Plasmids and BACs used in this work.

BAC	Description	Reference or source
2I16	The BAC containing 95 kb chromosomal fragment of *K. albida*; contains huimycin gene cluster	[1]
2I16_LS	The derivative of 2I16 with the deletion of 20 kb DNA fragment upstream the huiA gene	This work
2I16_RS	The derivative of 2I16 with the deletion of 36 kb DNA fragment downstream the KALB_4076 gene	This work
2I16_4069	The derivative of 2I16 with the deletion of KALB_4069 (*huiC*) gene	This work
2I16_4074	The derivative of 2I16 with the deletion of KALB_4074 gene	This work
2I16_4075	The derivative of 2I16 with the deletion of KALB_4075 gene	This work
2I16_4076	The derivative of 2I16 with the deletion of KALB_4076 gene	This work
pACS-hyg	The plasmid containing hygromycin resistance gene	[5]
pUC19	General cloning vector	Thermo Scientific

Table S3. Primers used in this study.

Primer	Sequence
LS_F	CGGTTCCGTCCTGTCACGAAGTGCGCCAGTTGGTAGAGGGCCGCGTGCGCAATACTTGACATATCACTGT
LS_R	TACGAGCCCAGCTCACTGAGGCTGGTCTCGAGTGTGTTGGGCGCGTCAGTTCTAGATCAGGCGCCGGGGGCGGTGT
RS_F	ACGCAGAGGCAGCCCGACGCGCCAGCCCACGGTCGTAGCAAGGAGCCAGGCGTCAGGTGGCACTTTTCG
RS_R	TGATGCAGCAGGGCACTGGGCAGGACGTAACTGGTTGACCGCAGGATCGCGATATCTTACCAATGCTTAATCAGTG
4069_F	ATGAGCATGGGAACATCCCGGACAGCGGTGACTCCTCTCGACGCCGAGGGTTTAAACCGTCAGGTGGCACTTTTCG
4069_R	CACAGCACCGAAGCCGTCGACGTGTGGGGGCGGGCGATCAGGACGGCGCCGTTTAAACTTACCAATGCTTAATCAGTG
4074_F	ATGATCGTTCTCGGACTGATCGGACGGCCCGACGTCCCGCTCTGCCATGGTTTAAACCGTCAGGTGGCACTTTTCG
4074_R	CAGGAGCGGCTCACCGCGTACGGCGGCATGACCAGCAGGTCGACGTCCGGGTTTAAACTTACCAATGCTTAATCAGTG
4075_F	ATGGGGAAGAGCTACGAGCGGATAGACGGCAGACTGCGTGCCTTCATCGGTTTAAACCGTCAGGTGGCACTTTTCG
4075_R	TATTCAGCGCGCGGAAGCGGTGGCAGCGGCAACGGCAACCCCGGCAGGCCGTTTAAACTTACCAATGCTTAATCAGTG
4076_F	ATGGTCGACAACATGCGGAGGTGTGATGAGATACCCGATTCCACGATTGGTTTAAACCGTCAGGTGGCACTTTTCG
4076_R	CAGGTCGGTGAGGTCGCTGTGGGGATTTCGGCGATACGCCAAACGCCGATGTTTAAACTTACCAATGCTTAATCAGTG
LS_chF	GTTCTCCCTTCCACCAGCC
LS_chR	TGTTCTTCAGGACGCGGAC
RS_chF	TGATTTCGTCGCGGTGGAA
RS_chR	GCCGCATACGACAGGGAAT
4069_chF	AACCTGCGGAACCTGCTAC
4069_chR	AACTTGCTGAGTCCCGCTC
4074_chF	CGAACAGTTGTGGTGTGCG
4074_chR	GGTCTTGACAATTGCTCCGG
4075_chF	CGATCCCCTTGGCATGTGA
4075_chR	CGCTGCTGGATGAGATCGT
4076_chF	AACCAACAAGGGGCTGTCC
4076_chR	AGTTCCACCGCGACGAAAT

References

1. Rebets, Y.; Tokovenko, B.; Lushchyk, I.; Rückert, C.; Zaburannyi, N.; Bechthold, A.; Kalinowski, J.; Luzhetskyy, A. Complete genome sequence of producer of the glycopeptide antibiotic Aculeximycin *Kutzneria albida* DSM 43870T, a representative of minor genus of *Pseudonocardiaceae*. *BMC Genomics* **2014**, *15*, 885, doi:10.1186/1471-2164-15-885.
2. Myronovskyi, M.; Rosenkränzer, B.; Nadmid, S.; Pujic, P.; Normand, P.; Luzhetskyy, A. Generation of a cluster-free *Streptomyces albus* chassis strains for improved heterologous expression of secondary metabolite clusters. *Metab. Eng.* **2018**, *49*, 316–324, doi:10.1016/j.ymben.2018.09.004.
3. Flett, F.; Mersinias, V.; Smith, C.P. High efficiency intergeneric conjugal transfer of plasmid DNA from *Escherichia coli* to methyl DNA-restricting streptomycetes. *FEMS Microbiol. Lett.* **1997**, *155*, 223–229, doi:10.1111/j.1574-6968.1997.tb13882.x.
4. Grant, S.G.; Jessee, J.; Bloom, F.R.; Hanahan, D. Differential plasmid rescue from transgenic mouse DNAs into *Escherichia coli* methylation-restriction mutants. *Proc. Natl. Acad. Sci. U.S.A.* **1990**, *87*, 4645–4649, doi:10.1073/pnas.87.12.4645.
5. Myronovskyi, M.; Brötz, E.; Rosenkränzer, B.; Manderscheid, N.; Tokovenko, B.; Rebets, Y.; Luzhetskyy, A. Generation of new compounds through unbalanced transcription of landomycin A cluster. *Appl. Microbiol. Biotechnol.* **2016**, *100*, 9175–9186, doi:10.1007/s00253-016-7721-3.

© 2020 by the authors. Licensee MDPI, Basel, Switzerland. This article is an open access article distributed under the terms and conditions of the Creative Commons Attribution (CC BY) license (http://creativecommons.org/licenses/by/4.0/).
